# Demographic disparities in the incidence and case fatality of subarachnoid haemorrhage: an 18-year nationwide study from New Zealand

**DOI:** 10.1016/j.lanwpc.2024.101199

**Published:** 2024-09-17

**Authors:** Ilari Rautalin, Rita V. Krishnamurthi, Craig S. Anderson, P. Alan Barber, Suzanne Barker-Collo, Derrick Bennett, Ronald Boet, Jason A. Correia, Jeroen Douwes, Andrew Law, Balakrishnan Nair, Amanda G. Thrift, Braden Te Ao, Bronwyn Tunnage, Anna Ranta, Valery Feigin

**Affiliations:** a The National Institute for Stroke and Applied Neurosciences, Auckland University of Technology, Auckland, New Zealand; b Department of Neurosurgery, University of Helsinki and Helsinki University Hospital, Helsinki, Finland; c The George Institute for Global Health, Faculty of Medicine, University of New South Wales, Kensington, Australia; d Department of Neurology, Royal Prince Alfred Hospital, Sydney, New South Wales, Australia; e Department of Medicine, Faculty of Medical and Health Sciences, The University of Auckland, Auckland, New Zealand; f Department of Neurology, Auckland City Hospital, Auckland, New Zealand; g School of Psychology, The University of Auckland, Auckland, New Zealand; h Clinical Trial Service Unit and Epidemiological Studies Unit, Nuffield Department of Population Health, University of Oxford, Oxford, UK; i Medical Research Council Population Health Research Unit, Nuffield Department of Population Health, University of Oxford, Oxford, UK; j Surgical Services, St. George's Hospital, Christchurch, New Zealand; k Department of Neurosurgery, Auckland City Hospital, Auckland, New Zealand; l Neurosurgery Research Unit, Centre for Brain Research, Faculty of Medical and Health Sciences, University of Auckland, Grafton, Auckland, New Zealand; m Research Centre for Hauora and Health, Massey University, Wellington, New Zealand; n Department of Medicine, School of Clinical Sciences at Monash Health, Monash University, Melbourne, VIC, Australia; o Health Systems, School of Population Health, Faculty of Medical and Health Sciences, University of Auckland, Auckland, New Zealand; p Department of Paramedicine, Auckland University of Technology, Auckland, New Zealand; q Department of Medicine, University of Otago, Wellington, New Zealand

**Keywords:** Case fatality, Ethnicity, Incidence, Regional variation, Subarachnoid haemorrhage, Temporal trends

## Abstract

**Background:**

Although the incidence and case-fatality of subarachnoid haemorrhage (SAH) vary within countries, few countries have reported nationwide rates, especially for multi-ethnic populations. We assessed the nationwide incidence and case-fatality of SAH in New Zealand (NZ) and explored variations by sex, district, ethnicity and time.

**Methods:**

We used administrative health data from the national hospital discharge and cause-of-death collections to identify hospitalised and fatal non-hospitalised aneurysmal SAHs in NZ between 2001 and 2018. For validation, we compared these administrative data to those of two prospective Auckland Regional Community Stroke Studies. We subsequently estimated the incidence and case-fatality of SAH and calculated adjusted rate ratios (RR) with 95% confidence intervals to assess differences between sub-populations.

**Findings:**

Over 78,187,500 cumulative person-years, we identified 5371 SAHs (95% sensitivity and 85% positive predictive values) resulting in an annual age-standardised nationwide incidence of 8.2/100,000. In total, 2452 (46%) patients died within 30 days after SAH. Compared to European/others, Māori had greater incidence (RR = 2.23 (2.08–2.39)) and case-fatality (RR = 1.14 (1.06–1.22)), whereas SAH incidence was also greater in Pacific peoples (RR = 1.40 (1.24–1.59)) but lesser in Asians (RR = 0.79 (0.71–0.89)). By domicile, age-standardised SAH incidence varied between 6.3–11.5/100,000 person-years and case fatality between 40 and 57%. Between 2001 and 2018, the SAH incidence of NZ decreased by 34% and the case fatality by 12%.

**Interpretation:**

Since the incidence and case-fatality of SAH varies considerably between regions and ethnic groups, caution is advised when generalising findings from focused geographical locations for public health planning, especially in multi-ethnic populations.

**Funding:**

NZ Health Research Council.

**Research in context**

**Evidence before this study**

We performed a literature search in the PubMed database to identify previous population-based cohort studies focused on the incidence and case-fatality of SAH as well as their differences by geographical regions, population groups, and time periods. We used the search terms: “subarachnoid haemorrhage AND (incidence OR epidemiology OR outcome OR mortality OR case-fatality) AND (population OR community OR epidemiology)” with no language or publication date restrictions. Based on the literature search, substantial variations exist in the incidence and case-fatality of SAH between countries and continents globally. However, as most nationwide evaluations rely on extrapolated figures from relatively small populations in defined geographical regions without continuous follow-ups, such estimates may be biased by within-country variations, as reported previously. Moreover, our literature search did not yield any long-term and nationwide population-based study that considered ethnic group differences while reporting nationwide incidence and case-fatality estimates for SAH.

**Added value of this study**

In this national study that included 5371 externally validated hospitalised and fatal non-hospitalised cases of SAH, we report, for the first time, nationwide figures for the incidence and case-fatality of SAH, overall and by time, sex, geographical district, and ethnic group in NZ. We found the incidence and case fatality differed substantially within NZ, with the largest rates observed among NZ Māori and people living in rural districts. Although the overall incidence and case-fatality of SAH decreased in NZ over time, some sub-populations and districts showed stable or even increasing secular trends.

**Implications of all the available evidence**

Incidence and case-fatality of SAH vary substantially for different ethnic subgroups, geographical regions, and time periods, within as well as across countries. Such heterogeneity should be considered in generalising regional findings for public health planning. Externally validated, nationwide administrative healthcare data collections may also be a useful source for monitoring the epidemiology of SAH, even in multi-ethnic countries. Since a similar approach might apply to other stroke types and acute neurological diseases, future studies around the topic are likewise warranted.

## Introduction

Systematic reviews of prospective population-based studies provide evidence that the incidence and case-fatality of subarachnoid haemorrhage (SAH) varies substantially between countries and over time.^1^^,^^2^ However, these nationwide estimates and between-country comparisons are primarily based on extrapolated figures from populations in small and defined, within-country geographical regions. Furthermore, the figures are typically applied across different population groups defined by age, sex, ethnicity, and region. Considering the prior evidence of substantial variations in the incidence and case-fatality of SAH between these sub-populations,^1^, ^2^, ^3^, ^4^, ^5^, ^6^ such generalisations may produce inaccurate estimates or even distort their use in recommendations for public health planning and resource allocation. According to recent studies from Finland,^3^^,^^5^, ^6^, ^7^ administrative healthcare data collections with external case validation are a valuable source for investigating national epidemiological measures of SAH, particularly in defining inter-regional and time differences. However, it is unclear whether these approaches apply to administrative data collections of other countries, especially those with multi-ethnic populations.

New Zealand (NZ) has a multi-ethnic population, with 70% self-identifying as NZ-European, 17% as Māori (the indigenous population of NZ), 15% as Asian, 8% as Pacific peoples, and 2% as Middle-Eastern, Latin American or African.^8^ However, studies of the incidence and case-fatality of SAH in NZ have primarily been based on population-based data from Greater Auckland, a single, ethnically diverse urban region in NZ.^4^^,^^9^^,^^10^ We aimed to externally validate two nationwide administrative health data collections including hospitalised and fatal non-hospitalised SAH events in NZ to determine national and sub-national, incidence and case-fatality of SAH by sex and ethnicity over 18 consecutive years. We hypothesised that such data vary substantially across time, geographical district, and ethnic group, having important implications for public health planning.

## Methods

To identify all cases of hospitalised and fatal non-hospitalised SAH in NZ between 2001 and 2018, we utilised two nationwide administrative data sources: the National Minimum Dataset (NMDS) and the Mortality Collection (MORT). This period was chosen because the data were available for both collections according to the International Classification of Disease 10th revision (ICD-10) codes and the NZ district health board (DHB) districts. Specifically, we used the NMDS to extract data about all publicly funded hospital discharges from 2001 to 2018 with primary or secondary diagnoses of SAH (I60.0–I60.9 according to the ICD-10 codes). In addition to diagnosis codes, we extracted data on hospitalisation admission and discharge dates, admission types, admission departments and hospital transfers. For fatal SAH cases, we linked the death data of MORT with hospital data of NMDS but also determined SAH cases (ICD-10 code I60.0–I60.9 as an underlying cause of death) who died without hospitalisation and had no NMDS data between 2000 and 2018. The data from 2000 were included to identify and exclude SAH deaths with a hospital visit during the preceding year of the study period. In addition to cause-of-death diagnoses, we extracted the death date, place of death, and information about any postmortem examination for all deaths from SAH. For all patients with SAH, we extracted routinely collected data on age, sex, prioritised ethnic group, and domicile at the time of SAH. In line with the general categorisation used by Statistics NZ,^11^ we categorised patients with SAH into four ethnic groups with the following prioritisation order: (i) Māori, (ii) Pacific peoples, (iii) Asian and (iv) European/other. Furthermore, we used the domicile codes to group cases according to the 20 DHBs of NZ, which correspond to the 20 sub-national districts of the country. To investigate whether the incidence and case-fatality of SAH depended on the rurality, we also divided the DHBs into urban and rural districts depending on whether they had a major urban area defined as a city with a population of more than 100,000 people.

We used the Auckland Regional COmmunity Stroke (ARCOS) Studies III and IV for external validation of our administrative case identification from NMDS/MORT. Details of these two prospective population-based cohorts have been described elsewhere.^12^^,^^13^ In brief, the studies were conducted in 2002–2003 (ARCOS III) and 2011–2012 (ARCOS IV) to identify all stroke events, including SAH, in the greater Auckland region comprising approximately one-third of the NZ population.^14^ Both cohorts incorporated multiple overlapping data sources, including inpatient, outpatient and discharge records, of all public and private hospitals in Auckland to identify hospitalised SAH cases. Autopsy reports, death certificates and coroners' reports were systematically ascertained to identify cases who died due to SAH without hospitalisation. Manual checks of prospective ‘hot pursuit’ data were made against administrative ‘cold pursuit’ data from the NZ Ministry of Health. Finally, the diagnosis of SAH for all included cases was verified by the Study Diagnostic Adjudication Committees consisting of stroke neurologists and geriatricians. All cases of SAH were confirmed by computerised tomography, magnetic resonance imaging or autopsy findings, whereas the aneurysmal origin of SAH was primarily confirmed via angiography, operation, or autopsy reports. As non-aneurysmal SAH is a heterogeneous group of diseases with varying aetiology, treatment and outcome that is also challenging to identify accurately via administrative data sources, our current administrative study solely focused on aneurysmal SAH cases. Therefore, we excluded angiogram-negative non-aneurysmal SAH cases identified via the ARCOS studies from the case validation. However, due to the negligible case fatality of non-aneurysmal SAHs,^15^ we also included hospitalised patients with SAH of ARCOS studies whose probable aneurysmal origin was not verified after brain computerised tomography because of immediate death or poor prognosis.

**Fig. 1:**
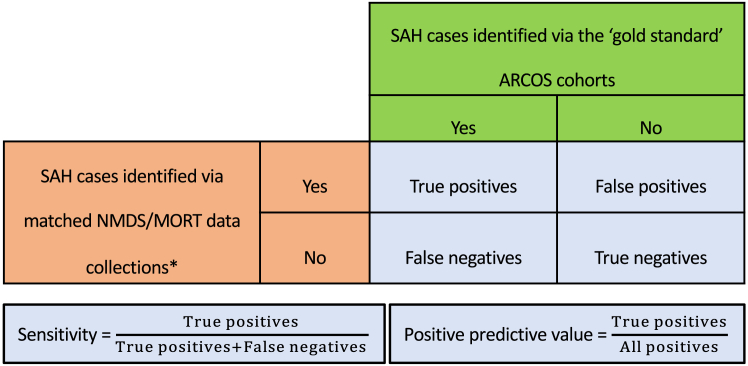
Definitions of sensitivity and positive predictive values for case validation between the cases of subarachnoid haemorrhage (SAH) identified in our administrative dataset from the National Minimum Dataset (NMDS) and the Mortality Collection (MORT) and the Auckland Regional Community Stroke Studies (ARCOS) that was used as the ‘gold standard’ reference cohort. ARCOS, Auckland Regional Community Stroke Studies; MORT, the Mortality Collection; NMDS, the National Minimum Dataset; SAH, subarachnoid haemorrhage. ∗For external case validation, we extracted the SAH cases from NMDS/MORT that occurred in the same time periods, age groups and geographical districts (according to the domicile of patients) as the ARCOS cohorts. These cases were matched with SAH cases identified via ARCOS cohorts according to their age, sex and date of SAH.

**Fig. 2:**
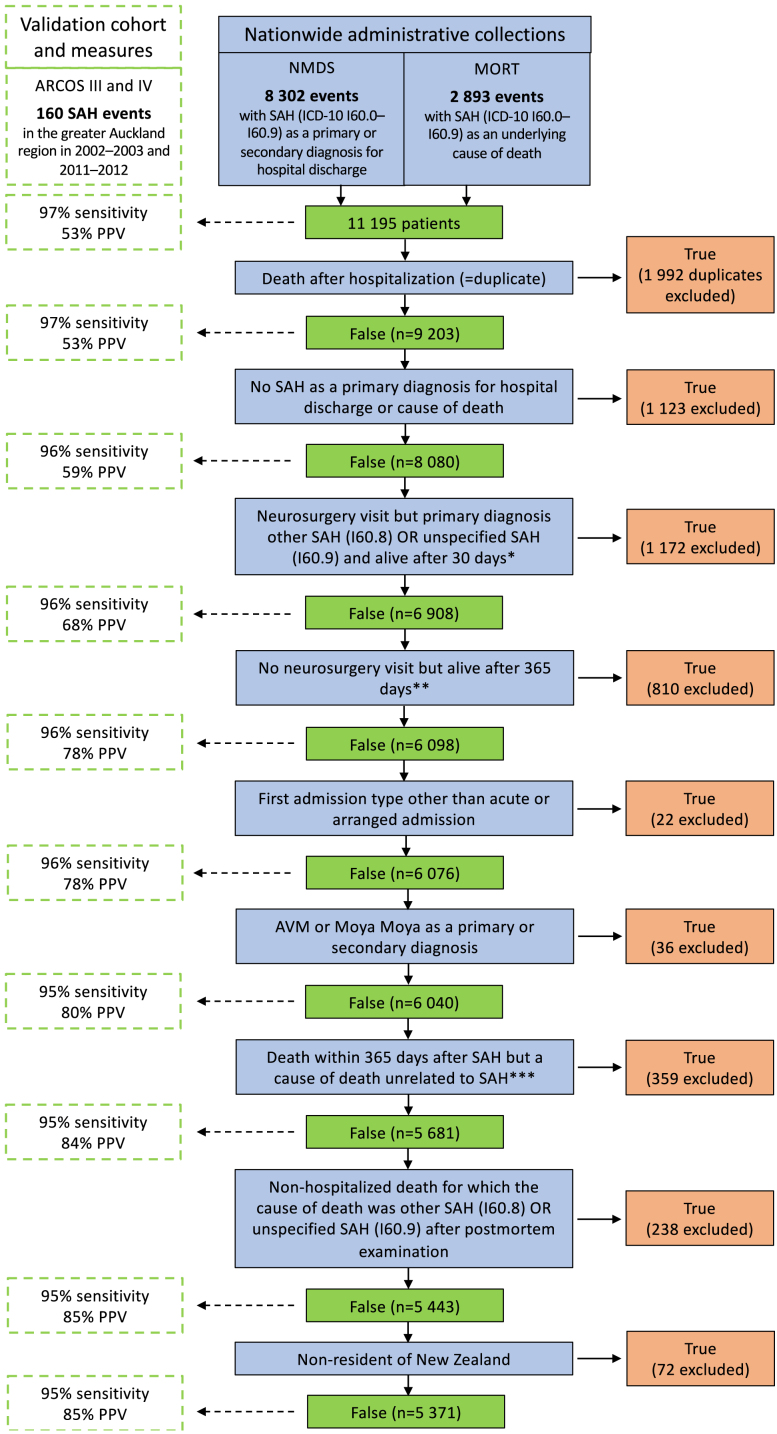
Details of the administrative case identification, stepwise modifications and validation measures. ARCOS, Auckland Regional Community Stroke Study; AVM, arteriovenous malformation; ICD-10, International Classification of Disease 10th revision; MORT, the Mortality Collection; NMDS, the National Minimum Dataset; PPV, positive predictive value; SAH, subarachnoid haemorrhage. ∗The aneurysmal origin of SAH is commonly verified via angiography and/or operation among patients who are admitted to a neurosurgery department with a relatively favourable prognosis indicating the operative approach. Therefore, we excluded cases that had unspecified SAH diagnoses (I60.8 or I60.9) even though they had been admitted to neurosurgery and were alive 30 days after SAH. These cases were most likely non-aneurysmal SAHs as none of the matched ‘true-positive’ SAH cases were excluded due to this modification. ∗∗Since the prognosis of true aneurysmal SAHs that are treated conservatively outside of the neurosurgical unit is extremely poor, we excluded events with no hospital visit in neurosurgery but who were alive 365 days after SAH. These cases were most likely conservatively treated non-aneurysmal SAHs as none of the matched ‘true-positive’ SAH cases were excluded due to this modification. ∗∗∗We excluded cases who died within 365 days after SAH where the stated cause of death was unrelated to SAH or its complications such as epileptic seizures, hydrocephalus, myocardial infarctions, heart arrhythmias, heart failure, pneumonia or other acute infections. These cases were most likely not true acute aneurysmal SAH because none of the matched ‘true-positive’ SAH cases were excluded due to this modification.

For the actual case validation, we compared the age-, time- and district-matched SAH cases from the administrative NMDS and MORT collections to identified cases via the ‘gold standard’ incidence cohorts of ARCOS. First, we extracted the SAH cases from NMDS and MORT who were adult (age ≥15 years in ARCOS III and ≥16 years in ARCOS IV) residents of the greater Auckland region in the periods of March 1, 2002 to February 28, 2003 (ARCOS III) and March 1, 2011 to February 28, 2012 (ARCOS IV). Second, we matched the extracted NMDS/MORT cases against reference SAH cases identified via ARCOS cohorts using the following criteria: (i) same sex, (ii) same age ±1-year difference to account for rounding error and (iii) same date of SAH admission/death ±10 days difference to account for potential multiple hospitalisations. This matching was done manually by determining the sex, age and date of SAH for each SAH case of the reference ARCOS cohorts and then verifying whether there was a counterpart in the administrative dataset. Third, we determined sensitivity and positive predictive values (PPV) for the NMDS/MORT-defined cases by comparing the number of matched cases (‘true positives’) to the overall SAH case numbers in the ARCOS validation cohorts and NMDS/MORT collections, respectively (Fig. 1). Finally, we utilised additional information about diagnosis codes, admission type and admission department to exclude probable false positive and non-aneurysmal SAH cases identified via the NMDS/MORT collections through modifications that preserved most true positives in the administrative data extraction (Fig. 2).

Following the modifications of the administrative data, we used the final study cohort to determine the general demographics of included SAH events in NZ between 2001 and 2018. For incidence calculations (defined as cases per 100,000 person-years with 95% confidence interval (CI)), we extracted the annual estimated resident populations of NZ from a publicly available database maintained by Statistics NZ.^11^ We also reported age-standardised incidence adjusted for the age distributions of the European standard population (ESP) in 2013 to enable closer comparisons to previous studies that are mostly from this regional population.^1^^,^^3^ For case-fatality, we calculated the proportion of patients with SAH who died either without hospitalisation or within 30 days following the first hospital visit of the event regardless of the cause of death. This definition was based on the previous epidemiological literature reporting the case fatality rates of SAH.^2^ To estimate temporal trends and compare the incidence and case-fatality of SAH in different districts and population groups, we used Poisson regression models to calculate age- and sex-adjusted rate ratios (RR) with 95% CIs. We also used these adjusted RRs to calculate age- and sex-adjusted case fatality for each DHB district by multiplying the crude case fatality of the Waitemata district (reference district with the largest population) by the district-specific RR. To control whether ethnic differences contributed to the geographic differences or temporal trends, we also computed separate ethnicity-adjusted regression analyses. In addition, we compared geographic incidence and case fatality rates separately in men and women, and in different ethnic groups. Lastly, in line with previous literature,^3^^,^^5^, ^6^, ^7^ we reported age-standardised incidence and crude 30-day case fatality during the first (2001–2003) and last three years (2016–2018) of the study period to present more readily interpretable secular figures. Stata version 17.0 (StataCorp 2021, College Station, TX, USA) was used for all analyses.

The NZ Ministry of Health (Reference: 2022-2312) and the Northern Health and Disability Ethics Committee of NZ (Reference: 2023 AM 9094) granted ethical approvals for our study. As the study was based on administratively collected and pseudonymised data, consent from patients with SAH was not required. The funders of the study had no role in the design and conduct of the study; in the collection, management, analysis, and interpretation of the data; or in the manuscript's preparation, review, or approval.

## Results

After excluding duplicates, our administrative case identification yielded 9203 cases with SAH (Fig. 2). In comparison to age-, time- and district-matched SAH cases from the NMDS and MORT, we found a match for 155 out of 160 SAH cases identified via our two subset validation cohorts of ARCOS resulting in 97% sensitivity. The PPV for the initial administrative extraction was 53%. After stepwise modifications, the PPV of our final study cohort rose to 85%, which was 92% in hospitalised SAH cases and 96% in patients with a hospital visit to neurosurgery (Fig. 2). Moreover, our final study cohort still comprised 95% of the SAH cases identified via the ARCOS validation cohorts.

During the total follow-up of 78,187,500 person-years (cumulative population of NZ between 2001 and 2018), our final study cohort comprised 5371 (67% women) patients with SAH (Table 1). The median age at the time of SAH was 57 years (interquartile range (IQR) 48–69). Of all patients with SAH, 69% identified as European/other, 19% as Māori, 6% as Asian and 5% as Pacific peoples. In comparison to European/other patients (median age 60 (50–72) years), Māori (median age 51 (43–59) years), Pacific peoples (median age 52 (44–63) years) and Asian (median age 56 (47–69) years) patients were younger at the time of SAH. Among all patients, 12% died without hospitalisation, 21% were treated conservatively outside of a neurosurgical unit, and 67% were admitted to a neurosurgical department.

**Table 1:** Average incidence and case-fatality of subarachnoid haemorrhage in New Zealand between 2001 and 2018 by sex and ethnicity.

	SAH events n (%)	Cumulative person-years n (%)	Crude incidence per 100,000 person-years (95% CI)	Age-standardized incidence per 100,000 person-years (95% CI)^a^	30-day case fatality in % (95% CI)
Overall					
All	5371 (100.0)	78,187,500 (100.0)	6.9 (6.7–7.1)	8.2 (8.0–8.4)	45.7 (44.4–47.0)
European/other	3691 (68.7)	52,665,360 (67.4)	7.0 (6.8–7.2)	7.2 (7.0–7.4)	47.3 (45.7–48.9)
Māori	1005 (18.7)	12,114,220 (15.5)	8.3 (7.8–8.8)	14.5 (13.8–15.2)	43.4 (40.3–46.5)
Pacific peoples	263 (4.9)	4,925,040 (6.3)	5.3 (4.7–6.0)	10.0 (9.1–10.9)	38.0 (32.1–43.9)
Asian	304 (5.7)	8,482,840 (10.8)	3.6 (3.2–4.0)	7.4 (6.8–8.0)	41.5 (36.0–47.0)
Missing^b^	108 (2.0)	–	–	–	41.7 (32.4–51.0)
Men					
All	1791 (100.0)	38,372,800 (100.0)	4.7 (4.5–4.9)	5.6 (5.4–5.8)	45.5 (43.2–47.8)
European/other	1254 (70.2)	25,859,970 (67.4)	4.8 (4.6–5.1)	5.1 (4.8–5.4)	45.8 (43.0–48.6)
Māori	316 (17.6)	5,947,140 (15.5)	5.3 (4.7–5.9)	9.7 (8.9–10.5)	45.9 (40.4–51.4)
Pacific peoples	89 (5.0)	2,445,530 (6.4)	3.6 (2.9–4.5)	6.6 (5.6–7.6)	43.8 (33.4–54.1)
Asian	92 (5.1)	4,119,990 (10.7)	2.2 (1.8–2.7)	4.2 (3.6–4.8)	45.7 (35.5–55.9)
Missing^b^	40 (2.2)	–	–	–	37.5 (22.5–52.5)
Women					
All	3580 (100.0)	39,814,600 (100.0)	9.0 (8.7–9.3)	10.5 (10.2–10.8)	45.7 (44.1–47.3)
European/other	2437 (68.1)	26,805,390 (67.3)	9.1 (8.7–9.5)	9.1 (8.7–9.5)	48.1 (46.1–50.1)
Māori	689 (19.3)	6,167,080 (15.5)	11.2 (10.4–12.0)	18.8 (17.7–19.9)	42.2 (38.5–45.9)
Pacific peoples	174 (4.9)	2,479,510 (6.2)	7.0 (6.0–8.1)	13.1 (11.7–14.5)	35.1 (28.0–42.2)
Asian	212 (5.9)	4,362,850 (11.0)	4.9 (4.2–5.6)	10.1 (9.2–11.0)	39.6 (33.0–46.2)
Missing^b^	68 (1.9)	–	–	–	44.1 (32.3–55.9)

CI, confidence interval; SAH, subarachnoid haemorrhage.

a Standardised to the age structure of the European Standard Population in 2013.

b Ethnicity one of the following: 1) don't know, 2) refused to answer, 3) response unidentifiable or 4) not stated.

The overall crude nationwide incidence of SAH in NZ was 6.9 (95% CI 6.7–7.1) per 100,000 person-years (Table 1). After age-standardisation to the 2013 ESP, the nationwide incidence rate was 8.2 (8.0–8.4) per 100,000 person-years. Of all patients, 2452 (46%) died within the first 30 days following SAH (Table 1). Based on age- and sex-adjusted regression models, women had approximately 82% (72–93%) greater incidence of SAH and 7% (1–12%) lesser case fatality than men. Compared to European/others, Māori (RR = 2.23 (2.08–2.39)) and Pacific peoples (RR = 1.40 (1.24–1.59)) had significantly greater, and Asians (RR = 0.79 (0.71–0.89)) significantly lesser SAH incidence. Moreover, the 30-day case fatality in Māori patients with SAH was higher (RR = 1.16 (1.07–1.26)) than in European/others. These differences between ethnic groups were similar in men and women (Supplementary Table S1).

**Fig. 3:**
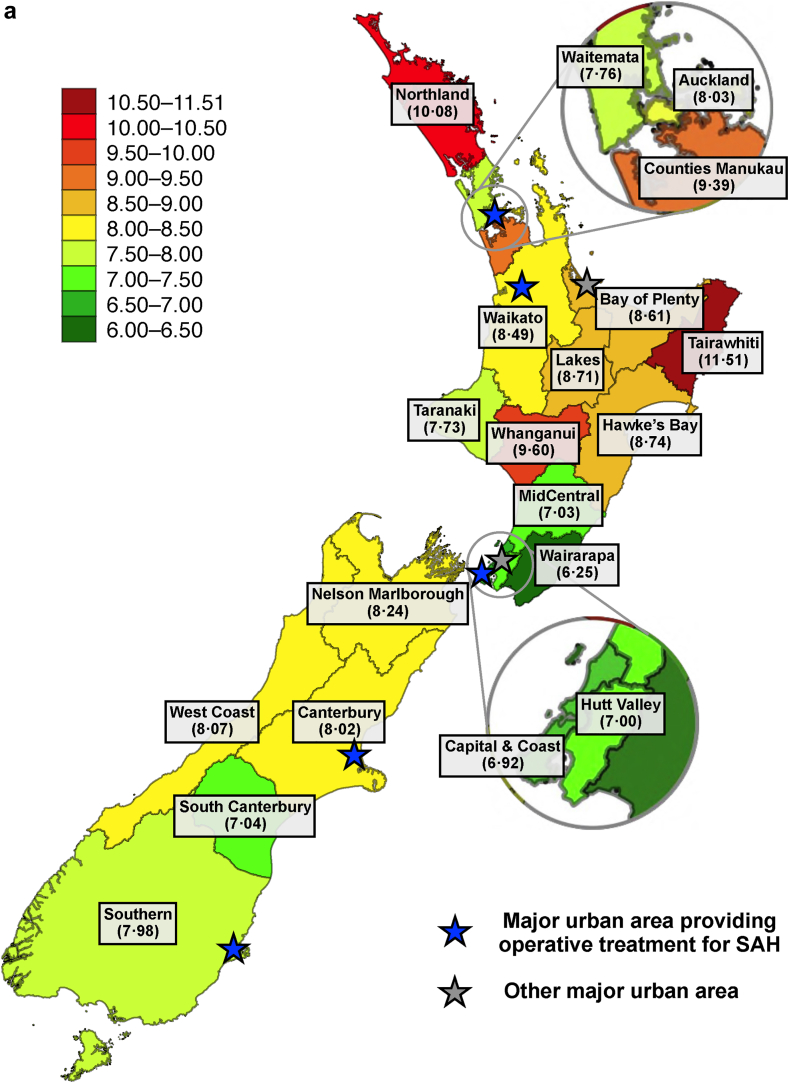

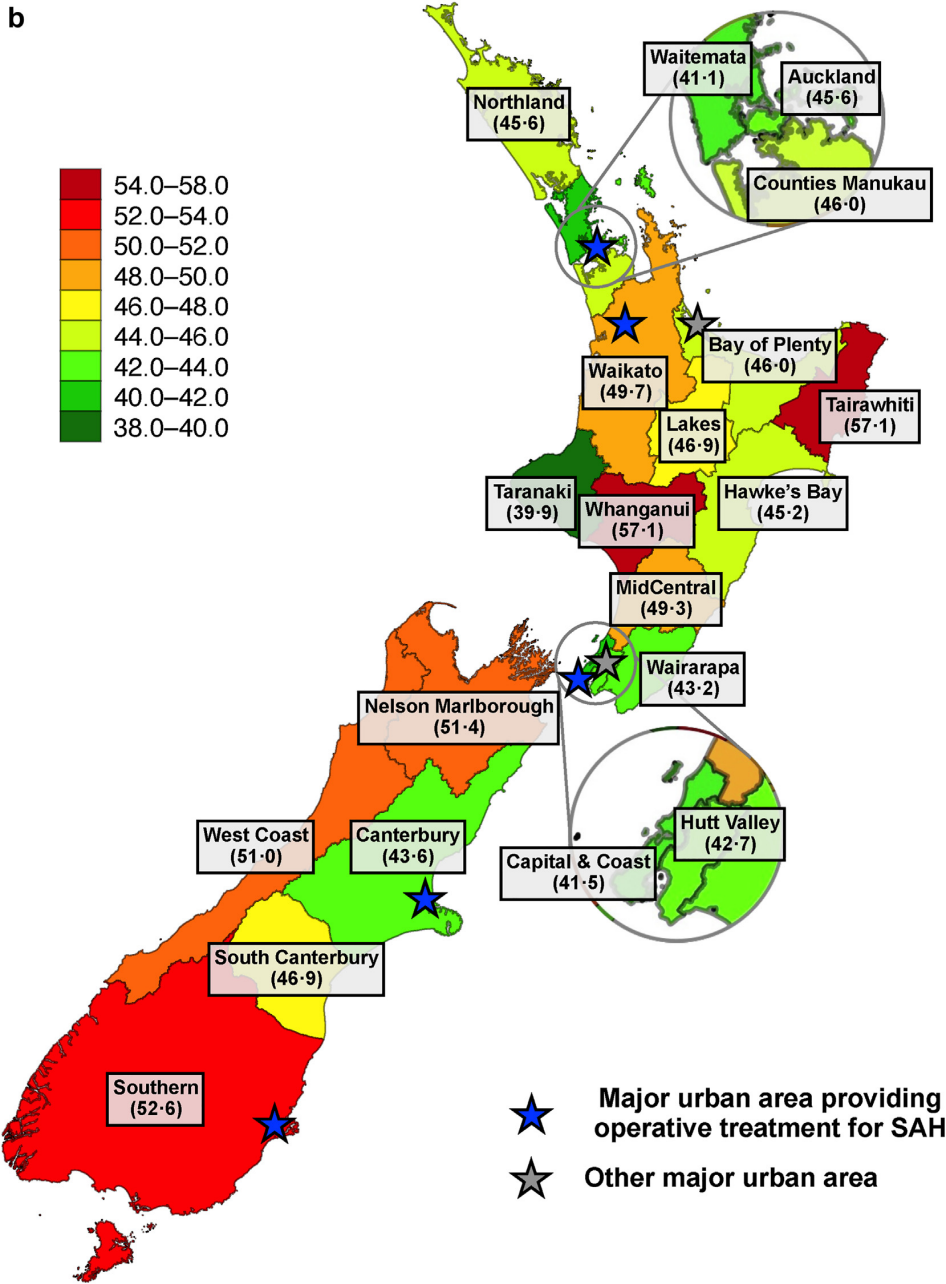
Average geographic A) age-standardised incidences of subarachnoid haemorrhage (SAH) (cases per 100,000 person-years) and B) age- and sex-adjusted 30-day case-fatality rates (%) of SAH in New Zealand between 2001 and 2018.

Incidence and case-fatality of SAH differed significantly between the 20 DHB districts of NZ (Fig. 3 and Table 2). The largest age-standardised incidence was found in Tairawhiti (11.5 per 100,000) and Northland (10.1 per 100,000), whereas the smallest incidence was observed in Wairarapa (6.3 per 100,000) and Hutt Valley (6.9 per 100,000) (Fig. 3A). In terms of age- and sex-adjusted case fatality, we observed the largest figures in Whanganui (57%) and Tairawhiti (57%), and the smallest figures in Taranaki (40%) and Waitemata (41%) (Fig. 3B). Both incidence and case-fatality of SAH were higher in rural than in urban districts (Table 2). These geographic differences were similarly observed in men and women (Supplementary Tables S2 and S3). After adjusting the regression models for geographical variation in ethnicity, we observed similar variations in case-fatality of SAH while the variations in incidence attenuated substantially (Table 2). Similarly, we found increased case fatality in many rural districts across all ethnic groups while the most substantial geographic differences in SAH incidence occurred in non-European ethnic groups, especially in the Māori population (Supplementary Table S4).

**Table 2:** Crude incidence and 30-day case-fatality rates as well as adjusted rate ratios with 95% confidence intervals for subarachnoid haemorrhage and its 30-day case fatality by geographical regions of NZ.

	SAH incidence			SAH case fatality		
	Crude incidence per 100,000 person-years (95% CI)	Partly-adjusted^a^ RR (95% CI)	Fully-adjusted^b^ RR (95% CI)	Crude 30-day case fatality in % (95% CI)	Partly-adjusted^a^ RR (95% CI)	Fully-adjusted^b^ RR (95% CI)
By DHBs						
Northland (R)	9.4 (8.3–10.5)	1.37 (1.18–1.58)	1.15 (0.99–1.33)	42.1 (36.2–48.0)	1.11 (0.94–1.30)	1.07 (0.91–1.25)
Waitemata (U)	6.2 (5.7–6.7)	[Reference]	[Reference]	41.1 (37.1–45.1)	[Reference]	[Reference]
Auckland (U)	5.9 (5.3–6.4)	1.02 (0.90–1.37)	1.02 (0.90–1.15)	43.6 (39.1–48.1)	1.06 (0.92–1.20)	1.06 (0.93–1.21)
Counties Manukau (U)	6.8 (6.2–7.3)	1.22 (1.09–1.37)	1.14 (1.01–1.28)	44.3 (40.3–48.3)	1.12 (0.99–1.28)	1.09 (0.96–1.25)
Waikato (U)	7.1 (6.5–7.7)	1.11 (0.98–1.25)	1.01 (0.89–1.14)	48.9 (44.4–53.4)	1.21 (1.06–1.37)	1.17 (1.03–1.34)
Lakes (R)	8.0 (6.7–9.3)	1.26 (1.06–1.52)	1.05 (0.88–1.26)	45.3 (37.3–53.3)	1.14 (0.94–1.38)	1.07 (0.88–1.30)
Bay of Plenty (U)	8.0 (7.1–8.9)	1.16 (1.01–1.33)	1.03 (0.89–1.18)	45.9 (40.3–51.5)	1.12 (0.97–1.30)	1.09 (0.94–1.26)
Tairawhiti (R)	9.6 (7.5–11.7)	1.58 (1.25–1.99)	1.17 (0.93–1.48)	51.9 (41.0–62.8)	1.39 (1.13–1.73)	1.30 (1.04–1.61)
Hawke's Bay (R)	7.8 (6.8–8.8)	1.14 (0.98–1.34)	1.02 (0.87–1.19)	44.3 (37.8–50.8)	1.10 (0.93–1.39)	1.06 (0.90–1.26)
Taranaki (R)	6.9 (5.7–8.0)	0.99 (0.82–1.19)	0.93 (0.77–1.12)	39.9 (31.7–48.1)	0.97 (0.79–1.20)	1.00 (0.81–1.23)
MidCentral (R)	6.1 (5.2–7.0)	0.97 (0.83–1.15)	0.91 (0.77–1.07)	51.0 (44.0–58.0)	1.20 (1.02–1.41)	1.19 (1.01–1.40)
Whanganui (R)	8.8 (7.0–10.5)	1.27 (1.02–1.57)	1.13 (0.91–1.39)	60.4 (50.9–69.9)	1.39 (1.17–1.66)	1.36 (1.14–1.62)
Capital & Coast (U)	5.4 (4.7–6.0)	0.88 (0.77–1.02)	0.86 (0.75–1.00)	40.9 (35.1–46.7)	1.01 (0.86–1.19)	1.01 (0.86–1.18)
Hutt Valley (U)	6.1 (5.1–7.0)	0.98 (0.82–1.16)	0.92 (0.77–1.10)	39.7 (32.0–47.4)	1.04 (0.84–1.27)	1.02 (0.83–1.25)
Wairarapa (R)	6.1 (4.4–7.9)	0.81 (0.59–1.10)	0.77 (0.56–1.04)	41.3 (27.1–55.5)	1.05 (0.76–1.46)	1.03 (0.73–1.45)
Nelson Marlborough (R)	7.9 (6.8–9.0)	1.06 (0.90–1.25)	1.05 (0.89–1.24)	54.0 (47.1–60.9)	1.25 (1.07–1.45)	1.26 (1.08–1.46)
West Coast (R)	7.6 (5.3–9.8)	1.06 (0.78–1.44)	1.04 (0.76–1.41)	52.3 (37.5–67.1)	1.24 (0.94–1.66)	1.24 (0.92–1.67)
Canterbury (U)	6.9 (6.3–7.4)	1.01 (0.90–1.13)	1.02 (0.91–1.14)	44.8 (40.9–48.7)	1.06 (0.93–1.19)	1.05 (0.93–1.19)
South Canterbury (R)	7.1 (5.4–8.7)	0.93 (0.73–1.19)	0.94 (0.73–1.20)	47.2 (35.7–58.7)	1.14 (0.89–1.46)	1.15 (0.90–1.47)
Southern (U)	7.0 (6.3–7.7)	1.06 (0.93–1.21)	1.05 (0.93–1.20)	52.9 (47.9–57.9)	1.28 (1.12–1.45)	1.29 (1.13–1.46)
By rurality						
Urban regions combined	6.6 (6.4–6.8)	[Reference]	[Reference]	44.9 (43.3–46.5)	[Reference]	[Reference]
Rural regions combined	7.8 (7.4–8.2)	1.08 (1.01–1.41)	1.01 (0.95–1.07)	47.5 (45.0–50.0)	1.06 (1.00–1.13)	1.05 (0.99–1.11)

CI, confidence interval; DHB, district health board; R, rural region; RR, rate ratio; SAH, subarachnoid haemorrhage; U, urban region.

a Partly-adjusted = adjusted for age and sex.

b Fully-adjusted = adjusted for age, sex, and ethnicity.

Between the first (2001–2003) and last (2016–2018) three years of the study period, the nationwide incidence of SAH decreased by 34% and 30-day case fatality by 12% (Table 3, Table 4). Based on age-, sex-, ethnicity- and district-adjusted regression models, we found an average annual decrease of 2.3% (95% CI 1.8–2.8%) in the incidence of SAH and 0.7% (0.2–1.2%) in the case-fatality of SAH between 2001 and 2018. The average annual decline in incidence was more pronounced in Europeans/others than in Māori, Pacific peoples or Asian groups (Table 3). For case fatality, we found declining trends in European, Pacific peoples and Asian patients, but stable rates in Māori patients (Table 4). Compared to the first three years of the study period, most districts had less incidence (19 out of 20 DHBs) and case-fatality (14 out of 20 DHBs) of SAH in the last three years (Table 3, Table 4). In contrast, four out of five districts in the South Island of NZ displayed increasing case fatality over the study period, although there was only a single significant increase in South Canterbury. In fact, during the last three years of our study period, all five districts of the South Island persisted with 30-day case fatality ≥50% and even as high as 75% in West Coast and 82% in South Canterbury (Table 4).

**Table 3:** Changes in the incidence of subarachnoid haemorrhage in New Zealand between 2001 and 2018 by sex, ethnicity and region.

	Age-standardised incidence of SAH per 100,000 person-years (95% CI)^a^		Adjusted RR (95% CI) between 2001–2003 and 2016–2018^b^	Annual decrease in % (95% CIs)^b^
	2001–2003	2016–2018		
Overall	10.8 (10.2–11.4)	7.1 (6.7–7.5)	0.66 (0.60–0.72)	2.3 (1.8–2.8)
By sex				
Men	7.2 (6.5–7.8)	5.1 (4.6–5.6)	0.72 (0.62–0.85)	1.9 (1.0–2.8)
Women	14.0 (13.1–15.0)	8.9 (8.2–9.6)	0.63 (0.56–0.71)	2.5 (1.9–3.2)
By ethnicity				
European/others	10.1 (9.4–10.7)	6.0 (5.5–6.4)	0.60 (0.54–0.67)	2.9 (2.3–3.6)
Māori	17.3 (15.4–19.2)	13.3 (11.9–14.8)	0.81 (0.66–1.00)	1.1 (−0.1 to 2.3)
Pacific peoples	8.3 (6.2–10.5)	9.0 (7.1–10.9)	1.22 (0.77–1.94)	−1.4 (−3.8 to 1.0)
Asian	10.4 (8.3–12.5)	5.9 (4.8–6.9)	0.77 (0.51–1.19)	1.1 (−1.2 to 3.3)
By region				
Northland (R)	12.5 (9.2–15.8)	10.1 (7.5–12.8)	0.82 (0.55–1.23)	1.7 (−0.6 to 3.9)
Waitemata (U)	10.8 (9.0–12.5)	7.1 (5.8–8.3)	0.71 (0.54–0.93)	2.0 (0.4–3.5)
Auckland (U)	10.3 (8.5–12.2)	6.3 (5.0–7.5)	0.63 (0.45–0.86)	2.8 (1.1–4.5)
Counties Manukau (U)	11.8 (9.9–13.7)	8.3 (6.9–9.7)	0.80 (0.60–1.06)	1.6 (0.0–3.2)
Waikato (U)	12.6 (10.4–14.8)	8.1 (6.5–9.7)	0.61 (0.45–0.82)	3.3 (1.6–5.0)
Lakes (R)	13.8 (9.6–18.0)	8.0 (5.0–11.0)	0.59 (0.34–1.01)	2.6 (−0.5 to 5.6)
Bay of Plenty (U)	12.5 (9.6–15.4)	9.1 (6.9–11.3)	0.70 (0.49–1.00)	2.2 (0.1–4.4)
Tairawhiti (R)	18.2 (11.0–25.3)	10.9 (5.5–16.2)	0.56 (0.28–1.11)	3.4 (−0.7 to 7.4)
Hawke's Bay (R)	12.9 (9.6–16.2)	5.0 (3.1–7.0)	0.38 (0.23–0.63)	4.8 (2.3–7.2)
Taranaki (R)	6.2 (3.5–9.0)	7.5 (4.6–10.3)	1.35 (0.71–2.58)	−1.2 (−4.6 to 2.1)
MidCentral (R)	7.8 (5.3–10.3)	6.6 (4.4–8.8)	0.83 (0.50–1.37)	0.7 (−2.1 to 3.4)
Whanganui (R)	10.9 (6.2–15.5)	6.1 (2.7–9.6)	0.56 (0.26–1.18)	2.9 (−0.8 to 6.6)
Capital & Coast (U)	8.4 (6.4–10.4)	6.3 (4.7–7.9)	0.75 (0.50–1.13)	1.6 (−0.7 to 3.8)
Hutt Valley (U)	8.4 (5.6–11.1)	7.5 (4.9–10.0)	0.88 (0.52–1.49)	−0.2 (−3.4 to 2.8)
Wairarapa (R)	7.8 (2.8–12.9)	3.2 (0.2–6.2)	0.44 (0.13–1.52)	0.6 (−5.3 to 6.1)
Nelson Marlborough (R)	9.7 (6.6–12.8)	7.2 (4.7–9.6)	0.75 (0.46–1.21)	1.4 (−1.3 to 4.1)
West Coast (R)	10.0 (3.6–16.4)	8.0 (2.4–13.6)	0.67 (0.24–1.88)	2.5 (−3.4 to 8.0)
Canterbury (U)	11.1 (9.4–12.9)	5.7 (4.5–6.8)	0.51 (0.38–0.69)	3.3 (1.7–4.8)
South Canterbury (R)	9.5 (4.8–14.2)	5.8 (2.3–9.3)	0.59 (0.27–1.30)	2.8 (−1.7 to 7.1)
Southern (U)	11.8 (9.5–14.1)	7.1 (5.4–8.8)	0.59 (0.43–0.82)	2.8 (0.9–4.7)
Urban regions combined	10.9 (10.3–11.6)	7.1 (6.6–7.6)	0.66 (0.59–0.73)	2.4 (1.8–3.0)
Rural regions combined	10.7 (9.5–11.8)	7.2 (6.3–8.1)	0.67 (0.56–0.80)	2.1 (1.1–3.0)

CI, confidence interval; R, rural region; RR, rate ratio; SAH, subarachnoid haemorrhage; U, urban region.

a Standardised to the age structure of the European Standard Population in 2013.

b Adjusted for age, sex, ethnicity and region.

**Table 4:** Changes in the 30-day case-fatality of subarachnoid haemorrhage in New Zealand between 2001 and 2018 by sex, ethnicity and region.

	Crude 30-day case fatality in % (95% CI)		Adjusted RR (95% CI) between 2001–2003 and 2016–2018^a^	Adjusted annual decrease in % (95% CI)^a^
	2001–2003	2016–2018		
Overall	49.0 (45.9–52.1)	44.0 (40.8–47.2)	0.88 (0.80–0.96)	0.7 (0.2–1.2)
By sex				
Men	50.0 (44.5–55.5)	44.9 (39.4–50.4)	0.88 (0.75–1.03)	0.7 (−0.2 to 1.6)
Women	48.5 (44.7–52.3)	43.6 (39.5–47.5)	0.88 (0.78–0.99)	0.7 (0.0–1.3)
By ethnicity				
European/others	50.5 (47.0–54.0)	45.6 (41.5–49.7)	0.86 (0.77–0.96)	0.8 (0.2–1.4)
Māori	44.0 (36.2–51.8)	45.9 (39.1–52.7)	0.99 (0.79–1.24)	−0.3 (−1.6 to 1.0)
Pacific peoples	37.0 (18.8–55.2)	29.6 (17.4–41.8)	0.85 (0.45–1.61)	1.1 (−2.1 to 4.2)
Asian	48.4 (30.8–66.0)	33.8 (22.8–44.8)	0.71 (0.43–1.17)	1.9 (−0.7 to 4.3)
By region				
Northland (R)	52.2 (37.8–66.6)	46.2 (32.6–59.8)	0.93 (0.64–1.33)	0.8 (−1.8 to 3.3)
Waitemata (U)	48.2 (38.9–57.5)	30.6 (22.0–39.2)	0.70 (0.51–0.97)	2.4 (0.7–4.0)
Auckland (U)	49.4 (38.9–59.9)	42.9 (31.3–54.5)	0.91 (0.65–1.27)	1.3 (−0.6 to 3.1)
Counties Manukau (U)	46.7 (36.5–56.9)	37.6 (28.5–46.7)	0.84 (0.60–1.18)	0.5 (−1.3 to 2.1)
Waikato (U)	52.6 (42.7–62.5)	51.7 (41.2–62.2)	0.89 (0.67–1.17)	0.7 (−1.0 to 2.4)
Lakes (R)	56.3 (39.1–73.5)	45.8 (25.9–65.7)	0.79 (0.47–1.33)	−0.2 (−3.8 to 3.2)
Bay of Plenty (U)	45.9 (33.4–58.4)	44.4 (32.1–56.7)	0.92 (0.64–1.33)	0.5 (−1.6 to 2.5)
Tairawhiti (R)	42.9 (21.7–64.1)	50.0 (23.8–76.2)	1.05 (0.55–2.01)	1.5 (−2.3 to 5.2)
Hawke's Bay (R)	45.9 (31.7–59.9)	37.5 (18.1–56.9)	0.83 (0.46–1.49)	−0.8 (−3.6 to 1.9)
Taranaki (R)	23.5 (3.3–43.7)	40.0 (20.8–59.2)	1.15 (0.46–2.88)	−2.9 (−7.0 to 1.1)
MidCentral (R)	46.7 (28.8–64.6)	42.4 (25.5–59.3)	0.90 (0.52–1.57)	1.5 (−1.1 to 4.0)
Whanganui (R)	58.8 (35.4–82.2)	50.0 (21.7–78.3)	0.92 (0.42–2.01)	−0.4 (−3.5 to 2.6)
Capital & Coast (U)	52.2 (37.8–66.6)	39.6 (25.8–53.4)	0.75 (0.50–1.14)	1.6 (−0.9 to 4.1)
Hutt Valley (U)	57.7 (38.7–76.7)	35.5 (18.7–52.3)	0.64 (0.36–1.14)	1.5 (−1.9 to 4.8)
Wairarapa (R)	62.5 (29.0–96.0)	25.0 (0.0–67.4)	0.36 (0.08–1.70)	1.7 (−5.4 to 8.3)
Nelson Marlborough (R)	47.1 (30.3–63.9)	52.9 (36.1–69.7)	0.92 (0.60–1.41)	0.3 (−1.9 to 2.5)
West Coast (R)	62.5 (29.0–96.0)	75.0 (45.0–100.0)	1.16 (0.53–2.54)	−1.4 (−7.5 to 4.4)
Canterbury (U)	44.2 (35.3–53.1)	53.8 (42.9–64.7)	0.97 (0.73–1.29)	0.2 (−1.4 to 1.9)
South Canterbury (R)	33.3 (9.4–57.2)	81.8 (59.0–100.0)	2.33 (1.05–5.15)	−3.2 (−7.6 to 0.9)
Southern (U)	56.3 (45.9–66.7)	50.0 (37.8–62.3)	0.88 (0.66–1.18)	0.8 (−0.9 to 2.4)
Urban regions combined	49.5 (45.9–53.1)	42.7 (38.9–46.5)	0.85 (0.76–0.95)	1.0 (0.4–1.6)
Rural regions combined	47.8 (41.9–53.7)	47.7 (41.4–54.0)	0.95 (0.80–1.13)	−0.1 (−1.0 to 0.9)

CI, confidence interval; R, rural region; RR, rate ratio; SAH, subarachnoid haemorrhage; U, urban region.

a Adjusted for age, sex, ethnicity and region.

## Discussion

This 18-year-long nationwide study of 5371 externally validated SAH cases from NZ showed that the incidence and case fatality of SAH varied substantially between different ethnic groups, geographical districts and time periods during the early 21st century. Specifically, the districts with the greatest rates had 96% larger incidence and 43% greater case fatality than the districts with the smallest rates while the differences were even more substantial after considering the variations between ethnic groups and time periods. These findings emphasise the importance of considering epidemiological variations of SAH even between population groups within countries. In other words, studies based on a single geographical region, short follow-up period and specific or unspecified ethnic groups may not accurately reflect national or other subgroup-specific estimates of SAH. Therefore, nationwide surveillance is needed for national and international public health planning and resource allocation for patients with SAH.

Similar to other studies of all stroke types,^4^^,^^9^ Pacific peoples and especially Māori populations had substantially greater SAH incidence than other ethnic groups. Since most geographic incidence differences disappeared after adjusting for ethnicity, differences in the geographic distribution by ethnic groups may at least partly explain the incidence differences between districts. In addition, the geographic incidence differences occurred primarily in non-European ethnic groups. One explanation for differences by ethnicity may relate to the prevalence of cigarette smoking, which is a key risk factor for SAH.^16^, ^17^, ^18^ For example, the three NZ census data collections that occurred during our study period found that smoking rates in Māori (33%) and Pacific people groups (24%) were more than 2- and 1.5-fold larger than that in European/other populations (15%).^19^ Similarly, the three districts with the largest age-standardised incidence of SAH had the greatest prevalence of smoking.^19^ Similar to the changes in the incidence of SAH, nationwide smoking rates in NZ have decreased by 36% (from 21% to 13%) between 2006 and 2018, and these trends were more pronounced in European/other (37%) and Asian (38%) than in the Māori (33%) and Pacific peoples (30%) populations.^19^ Nevertheless, while smoking likely plays a significant role, we have insufficient data to assess the potential impact of other ethnicity-related risk factors and the cause of the observed variations is likely complex and multi-factorial.

Regarding potential contributors to differences in case-fatality, the districts with the largest case-fatality of SAH are rural and have some of the longest transportation times in NZ to reach advanced emergency medical care.^20^ As the definitive treatment of SAH in NZ is centralised into five urban public university hospitals with a neurosurgical unit (Fig. 3), patients with SAH from rural districts may also have the longest delays in hospital admission and operative treatment. In addition, part of the geographic variation may relate to the differences in treatment modalities and their availability between neurosurgical units of NZ. For example, the neurosurgical unit in the Southern DHB, a district with the third highest case fatality, does not provide endovascular services. Therefore, patients with SAH from most districts of the South Island of NZ who require endovascular operations are primarily transferred to the neurosurgical unit in Canterbury. Regarding differences by ethnicity, similar delays to hospitalisation have also been reported in the Māori population,^20^ which have been attributed to communication, cultural and financial barriers leading to compromised access to hospital services.^21^ Moreover, Māori patients with SAH appear to suffer more commonly from expansive bleeding and delayed cerebral ischaemia which are related to poor SAH outcomes.^22^ In terms of secular trends, declining case-fatality in most districts and ethnic groups may be attributable to the greater availability of acute brain imaging,^23^ shortened treatment delay,^2^ advances in multidisciplinary neurocritical care,^24^ novel endovascular aneurysm securing modalities,^25^ and decreasing prevalence of smoking.^26^ On the other hand, the case fatality in certain districts, particularly in the South Island of NZ, increased over time to an alarmingly high level requiring further local monitoring, especially considering that the South Island of NZ is reported to have a severe shortage of neurosurgeons.^27^ Furthermore, future studies focusing on the prehospital death rates and case fatality rates among hospitalised SAH patients in different population groups and geographical regions of NZ are warranted.

Our findings in general are consistent with previous population-based studies from NZ and internationally.^1^^,^^2^^,^^5^^,^^6^^,^^28^ However, apart from a few previous Finnish studies,^3^^,^^5^, ^6^, ^7^ we are not aware of other nationwide population-based studies of incidence and case-fatality of SAH based on externally validated administrative hospital discharge and cause-of-death data. In comparison to Finnish studies,^3^^,^^5^, ^6^, ^7^ we observed similar case identification validity, nationwide age-standardised incidence, crude case-fatality and decreasing trends of SAH in the early 21st century (Supplementary Table S5). However, in NZ, the sub-national geographic differences in both incidence and case-fatality of SAH were more than two-fold greater than in Finland which may relate to the greater ethnically and socioeconomically diverse population of NZ. On the other hand, this difference may also relate to our more extensive geographical categorisation based on 20 different districts in NZ whereas the Finnish studies only comprised five university hospital catchment areas, none of which would have been categorised as rural regions according to our definitions. Nevertheless, considering the high sensitivity and PPV of these administrative studies, our findings provide evidence that administrative hospital discharge and cause-of-death data can serve as a useful platform to investigate the epidemiological measures of SAH and potentially other stroke types that require urgent hospital admission. However, due to the large number of false positive hospitalisations and non-hospitalised sudden deaths, case identification in such studies should always be externally validated.

Despite the strengths of a nationwide design, large and representative sample size, long-term follow-up, comprehensive subgroup analyses and an almost complete identification of true SAH events in the reference cohorts with only a fraction of missing data on collected patient characteristics, our study has limitations. First, although the accuracy of our case identification for hospitalised SAH cases was high, the PPV for sudden-death SAH was only 26%. However, since the aneurysmal SAH diagnosis (I60.0–I60.7) for most (79%) of these ‘false positives’ was verified by postmortem examination and case-fatality of non-aneurysmal SAH is negligible,^15^ we believe that most of these ‘false positives’ were actually true aneurysmal SAH and thus, we did not exclude them. As a possible explanation for the discrepancy between the sudden-death identification of the administrative datasets and reference ARCOS cohorts, some of the true sudden-death SAH may have been identified after the data collection of the reference ARCOS cohorts, since the final version of the MORT was completed after a five-year delay. Second, we were only able to validate the SAH cases in the greater Auckland region during two individual 12-month periods. While we are unaware that the administrative collection methods for SAH vary between different geographical districts of NZ or over time and thus this limitation is unlikely to have significantly influenced our main conclusions, it would be useful to conduct additional validation studies in other parts of the country as part of future projects. Third, since not all sudden deaths undergo postmortem examination in NZ, we may have missed some sudden-death SAHs. This can also at least partly explain why our sudden death rate (12%) was significantly less than the previously reported rate in Finland (24%),^5^ where the general autopsy rate is greater. Nevertheless, because patients with SAH are often healthy and relatively young, leading to autopsy also in NZ, we believe that the number of missed SAH events is likely smaller than the difference in sudden-death rate would indicate. Moreover, as the autopsy rates have not decreased over the study period and do not vary substantially between ethnic groups and presumably between geographical districts of NZ (Supplementary Figure S1), we believe that any underestimations are minor and unlikely to invalidate our conclusions. Finally, since our study cohort was based on two administrative data sources, it did not include individual-level data about many patient- or SAH-related characteristics that would provide more information about the causes of observed differences in incidence and case fatality of SAH as well as their time trends. Moreover, we did not have information whether the number of operations for SAH or unruptured intracranial aneurysms (UIAs) varied between population groups and geographic districts or changed over the study period. Since people from the population groups (e.g., minor ethnic groups) and regions (e.g., rural districts) with the highest incidence and case fatality of SAH may also seek more rarely medical assistance due to unrelated reasons, it is possible that their ‘high-risk’ incidental UIAs are not detected and preventatively treated as commonly as in subpopulation with lower risk of SAH. Therefore, future studies with comprehensive data on chronic illnesses including UIAs, socioeconomic status, lifestyle-related risk factors, diagnostic/treatment modalities, in-hospital complications and cause-specific deaths among patients with SAH are needed to establish countrywide recommendations for programs to prevent and improve outcomes from SAH in NZ.

In summary, we estimated the age-standardised nationwide SAH incidence of 8.2 per 100,000 person-years and a case-fatality of 46% in NZ. Between 2001 and 2018, these estimates decreased by 34% for incidence and 12% for case fatality. However, because incidence and case-fatality of SAH appear to vary substantially between geographical regions and ethnic groups, future studies should carefully consider such differences, especially when comparing the figures of different countries and population groups.

## Contributors

IR, RK, AR and VF contributed to the design of the manuscript. IR drafted the first version of the manuscript but all authors revised the manuscript critically. IR performed all statistical analyses of the study but all authors participated in interpreting the results. IR and BN verified the underlying data. All authors approved the final version of the manuscript and its submission.

## Data sharing statement

Due to local ethics requirements, we cannot share the data for public use. However, qualified researchers can apply for access to the used datasets sourced from the NZ Ministry of Health. Furthermore, more information about the detailed study protocol and statistical analysis plan can be obtained from the corresponding author.

## Editor note

The Lancet Group takes a neutral position with respect to territorial claims in published maps and institutional affiliations.

## Declaration of interests

IR received research grants from the Sigrid Juselius Foundation, the Finnish Medical Foundation, the Sakari Alhopuro Foundation, the Finnish Foundation for Cardiovascular Research and the Maud Kuistila Foundation. AR received grant funding from the New Zealand Health Research Council and the Ministry of Health. JD is funded from grants awarded by the New Zealand Health Research Council and the New Zealand Royal Society Marsden Fund. PAB report a leadership or fiduciary role as a President of Australia and New Zealand Association of Neurologists. The authors report no other declarations.
