# Supplementary material for: Demographic disparities in the incidence and case fatality of subarachnoid haemorrhage: an 18-year nationwide study from New Zealand
Source: Lancet Reg Health West Pac. 2024 Sep 17;52:101199. doi: 10.1016/j.lanwpc.2024.101199 (PMC11422095; doi:10.1016/j.lanwpc.2024.101199)
Supplement: Supplementary Tables and Figure [file mmc1.docx]

# **Supplementary Files**

**Supplementary Table 1.** Age-adjusted rate ratios with 95% confidence intervals for the incidence and 30-day case fatality of subarachnoid haemorrhage in New Zealand between 2001 and 2018 by ethnic group and sex.

**Supplementary Table 2.** Crude incidence rates and adjusted incidence rate ratios with 95% confidence intervals for subarachnoid haemorrhage by sex and the geographical regions of New Zealand.

**Supplementary Table 3.** Crude case-fatality rates and adjusted rate ratios with 95% confidence intervals for 30-day case-fatality after subarachnoid haemorrhage by sex and the geographical regions of New Zealand.

**Supplementary Table 4.** Age- and sex-adjusted rate ratios with 95% confidence intervals for subarachnoid haemorrhage and its 30-day case fatality by ethnicity and geographical regions of NZ.

**Supplementary Table 5.** Comparison between the current study from New Zealand and the previous studies from Finland.

**Supplementary Figure 1A–G.** Annual autopsy rates for all out-of-hospital deaths (facility code of death was 'unknown' or '9990') in NZ by age groups, sex and ethnic groups between 2001 and 2018. Data were requested from the NZ Ministry of Health and are based on the Mortality Collection.

**Supplementary Table 1.** Age-adjusted rate ratios with 95% confidence intervals for the incidence and 30-day case fatality of subarachnoid haemorrhage in New Zealand between 2001 and 2018 by ethnic group and sex.

| Ethnic group | Men | Women | Overall |
| --- | --- | --- | --- |
|  | Age-adjusted RR with 95% CI for SAH | | |
| European/other | [Reference] | [Reference] | [Reference] |
| Māori | 2·00 (1·77–2·27) | 2·35 (2·15–2·56) | 2·25 (2·10–2·42) |
| Pacific peoples | 1·32 (1·06–1·64) | 1·45 (1·24–1·69) | 1·41 (1·24–1·60) |
| Asian | 0·70 (0·57–0·87) | 0·84 (0·73–0·97) | 0·81 (0·72–0·91) |
|  | Age-adjusted RR with 95% CI for 30-day fatality after SAH | | |
| European/other | [Reference] | [Reference] | [Reference] |
| Māori | 1·23 (1·07–1·40) | 1·13 (1·02–1·25) | 1·16 (1·07–1·26) |
| Pacific peoples | 1·17 (0·92–1·49) | 0·87 (0·71–1·07) | 0·97 (0·83–1·13) |
| Asian | 1·11 (0·89–1·37) | 0·89 (0·76–1·05) | 0·95 (0·84–1·08) |

CI = confidence interval; RR = rate ratio; SAH = subarachnoid haemorrhage

**Supplementary Table 2.** Crude incidence rates and adjusted incidence rate ratios with 95% confidence intervals for subarachnoid haemorrhage by sex and the geographical regions of New Zealand.

| Region | Men | | | Women | | |
| --- | --- | --- | --- | --- | --- | --- |
|  | Crude incidence  (per 100,000) | Partly adjusted* RR  (95% CI) | Fully adjusted** RR  (95% CI) | Crude incidence  (per 100,000) | Partly adjusted* RR  (95% CI) | Fully adjusted** RR  (95% CI) |
| By DHB |  |  |  |  |  |  |
| Northland (R) | 6·9 (5·5–8·2) | 1·50 (1·17–1·91) | 1·28 (1·00–1·64) | 11·9 (10·1–13·7) | 1·31 (1·09–1·56) | 1·08 (0·90–1·30) |
| Waitemata (U) | 4·2 (3·6–4·7) | [Reference] | [Reference] | 8·2 (7·4–9·0) | [Reference] | [Reference] |
| Auckland (U) | 3·8 (3·2–4·5) | 0·98 (0·79–1·22) | 1·00 (0·81–1·24) | 7·8 (7·0–8·7) | 1·03 (0·89–1·20) | 1·03 (0·89–1·20) |
| Counties Manukau (U) | 4·9 (4·2–5·5) | 1·29 (1·05–1·57) | 1·23 (1·00–1·50) | 8·6 (7·8–9·5) | 1·19 (1·04–1·38) | 1·09 (0·95–1·26) |
| Waikato (U) | 4·8 (4·0–5·6) | 1·11 (0·90–1·38) | 1·02 (0·82–1·26) | 9·3 (8·3–10·4) | 1·10 (0·95–1·28) | 1·00 (0·86–1·16) |
| Lakes (R) | 5·8 (4·2–7·3) | 1·37 (1·01–1·86) | 1·17 (0·86–1·59) | 10·2 (8·2–12·2) | 1·21 (0·97–1·52) | 1·00 (0·79–1·25) |
| Bay of Plenty (U) | 5·5 (4·4–6·5) | 1·20 (0·94–1·53) | 1·07 (0·84–1·37) | 10·5 (9·0–11·9) | 1·14 (0·96–1·35) | 1·01 (0·85–1·20) |
| Tairawhiti (R) | 6·1 (3·7–8·4) | 1·51 (0·99–2·29) | 1·17 (0·77–1·78) | 13·0 (9·6–16·4) | 1·62 (1·22–2·14) | 1·17 (0·88–1·55) |
| Hawke's Bay (R) | 4·8 (3·6–6·0) | 1·10 (0·83–1·45) | 0·98 (0·74–1·30) | 10·7 (9·0–12·4) | 1·17 (0·97–1·41) | 1·03 (0·86–1·25) |
| Taranaki (R) | 4·6 (3·3–6·0) | 0·99 (0·71–1·38) | 0·92 (0·66–1·29) | 9·0 (7·2–10·9) | 0·98 (0·78–1·24) | 0·93 (0·74–1·17) |
| MidCentral (R) | 4·5 (3·4–5·6) | 1·05 (0·80–1·39) | 0·99 (0·74–1·30) | 7·6 (6·2–9·0) | 0·93 (0·76–1·14) | 0·88 (0·72–1·08) |
| Whanganui (R) | 6·9 (4·7–9·1) | 1·55 (1·10–2·18) | 1·38 (0·98–1·95) | 10·6 (7·9–13·2) | 1·13 (0·87–1·49) | 1·01 (0·77–1·32) |
| Capital & Coast (U) | 3·2 (2·5–4·0) | 0·81 (0·63–1·05) | 0·79 (0·61–1·03) | 7·3 (6·3–8·4) | 0·92 (0·77–1·09) | 0·90 (0·75–1·07) |
| Hutt Valley (U) | 4·7 (3·5–5·8) | 1·11 (0·83–1·49) | 1·05 (0·78–1·40) | 7·4 (5·9–8·9) | 0·91 (0·73–1·14) | 0·86 (0·69–1·07) |
| Wairarapa (R) | 5·2 (2·9–7·5) | 1·09 (0·68–1·74) | 1·02 (0·64–1·64) | 7·0 (4·4–9·7) | 0·68 (0·45–1·01) | 0·64 (0·43–0·96) |
| Nelson Marlborough (R) | 6·3 (4·9–7·7) | 1·26 (0·96–1·64) | 1·22 (0·93–1·60) | 9·5 (7·8–11·2) | 0·97 (0·79–1·19) | 0·97 (0·79–1·19) |
| West Coast (R) | 5·8 (3·0–8·5) | 1·20 (0·73–1·97) | 1·17 (0·71–1·92) | 9·4 (5·9–13·0) | 0·99 (0·66–1·47) | 0·97 (0·65–1·44) |
| Canterbury (U) | 4·3 (3·7–4·9) | 0·96 (0·78–1·18) | 0·95 (0·77–1·16) | 9·4 (8·5–10·3) | 1·04 (0·90–1·19) | 1·05 (0·92–1·21) |
| South Canterbury (R) | 5·4 (3·3–7·4) | 1·06 (0·71–1·60) | 1·05 (0·70–1·59) | 8·7 (6·2–11·3) | 0·87 (0·64–1·19) | 0·88 (0·65–1·21) |
| Southern (U) | 4·4 (3·6–5·2) | 0·99 (0·78–1·24) | 0·96 (0·76–1·22) | 9·6 (8·4–10·7) | 1·10 (0·94–1·28) | 1·10 (0·94–1·29) |
| By rurality |  |  |  |  |  |  |
| Urban regions combined | 4·4 (4·1–4·6) | [Reference] | [Reference] | 8·7 (8·3–9·0) | [Reference] | [Reference] |
| Rural regions combined | 5·6 (5·1–6·1) | 1·18 (1·06–1·30) | 1·11 (1·00–1·23) | 9·9 (9·3–10·5) | 1·03 (0·95–1·11) | 0·96 (0·89–1·03) |

CI = confidence interval; DHB = district health board; R = rural region; RR = rate ratio; U = urban region

*Partly adjusted = adjusted for age

**Fully adjusted = adjusted for age and ethnicity

**Supplementary Table 3.** Crude 30-day case-fatality rates and adjusted rate ratios with 95% confidence intervals for 30-day case-fatality after subarachnoid haemorrhage by sex and the geographical regions of New Zealand.

| Region | Men | | | Women | | |
| --- | --- | --- | --- | --- | --- | --- |
|  | Crude CFR in % | Partly adjusted* RR (95% CI) | Fully adjusted** RR (95% CI) | Crude CFR in % | Partly adjusted* RR (95% CI) | Fully adjusted** RR (95% CI) |
| By DHBs |  |  |  |  |  |  |
| Northland (R) | 44·9 (35·1–54·7) | 1·22 (0·94–1·57) | 1·19 (0·91–1·56) | 40·6 (33·3–47·9) | 1·05 (0·89–1·23) | 1·00 (0·82–1·23) |
| Waitemata (U) | 38·7 (31·8–45·6) | [Reference] | [Reference] | 42·2 (37·3–47·1) | [Reference] | [Reference] |
| Auckland (U) | 41·1 (33·3–48·9) | 1·10 (0·86–1·40) | 1·08 (0·85–1·39) | 44·8 (39·3–50·3) | 1·05 (0·89–1·23) | 1·05 (0·90–1·23) |
| Counties Manukau (U) | 48·3 (41·5–55·1) | 1·25 (1·01–1·54) | 1·18 (0·94–1·47) | 42·2 (37·2–47·2) | 1·06 (0·91–1·24) | 1·05 (0·90–1·24) |
| Waikato (U) | 48·7 (40·9–56·5) | 1·26 (1·00–1·57) | 1·24 (0·98–1·56) | 49·0 (43·5–54·5) | 1·18 (1·01–1·37) | 1·14 (0·98–1·34) |
| Lakes (R) | 45·3 (31·9–58·7) | 1·19 (0·87–1·65) | 1·16 (0·83–1·61) | 45·4 (35·5–55·3) | 1·12 (0·89–1·40) | 1·02 (0·80–1·32) |
| Bay of Plenty (U) | 41·6 (32·0–51·2) | 1·05 (0·80–1·39) | 1·02 (0·77–1·35) | 48·0 (41·1–54·9) | 1·15 (0·96–1·36) | 1·12 (0·95–1·33) |
| Tairawhiti (R) | 48·0 (28·4–67·6) | 1·32 (0·90–1·96) | 1·24 (0·81–1·89) | 53·6 (40·5–66·7) | 1·39 (1·10–1·76) | 1·32 (1·02–1·69) |
| Hawke's Bay (R) | 42·4 (30·5–54·3) | 1·10 (0·80–1·50) | 1·07 (0·79–1·46) | 45·2 (37·4–53·0) | 1·09 (0·90–1·33) | 1·06 (0·87–1·29) |
| Taranaki (R) | 34·8 (21·0–48·6) | 0·85 (0·56–1·31) | 0·90 (0·59–1·35) | 42·4 (32·3–52·5) | 1·02 (0·80–1·31) | 1·04 (0·82–1·32) |
| MidCentral (R) | 53·0 (41·0–65·0) | 1·27 (0·95–1·69) | 1·24 (0·95–1·61) | 50·0 (41·3–58·7) | 1·18 (0·96–1·44) | 1·16 (0·94–1·42) |
| Whanganui (R) | 59·0 (43·6–74·4) | 1·43 (1·05–1·96) | 1·41 (1·03–1·92) | 61·3 (49·2–73·4) | 1·42 (1·13–1·79) | 1·34 (1·08–1·65) |
| Capital & Coast (U) | 39·5 (28·9–50·1) | 1·00 (0·74–1·36) | 0·99 (0·74–1·33) | 41·5 (34·6–48·4) | 1·02 (0·84–1·24) | 1·02 (0·84–1·24) |
| Hutt Valley (U) | 42·4 (29·8–55·0) | 1·15 (0·84–1·58) | 1·13 (0·81–1·58) | 38·1 (28·4–47·8) | 0·97 (0·76–1·26) | 0·95 (0·73–1·24) |
| Wairarapa (R) | 47·4 (24·9–69·9) | 1·18 (0·73–1·93) | 1·19 (0·74–1·93) | 37·0 (18·8–55·2) | 0·95 (0·61–1·50) | 0·92 (0·56–1·53) |
| Nelson Marlborough (R) | 53·9 (42·8–65·0) | 1·29 (0·99–1·68) | 1·36 (1·05–1·75) | 54·2 (45·3–63·1) | 1·23 (1·01–1·50) | 1·21 (1·00–1·46) |
| West Coast (R) | 52·9 (29·2–76·6) | 1·36 (0·86–2·14) | 1·42 (0·88–2·28) | 51·9 (33·1–70·7) | 1·18 (0·81–1·72) | 1·16 (0·79–1·70) |
| Canterbury (U) | 46·6 (39·5–53·7) | 1·17 (0·93–1·46) | 1·15 (0·92–1·45) | 44·0 (39·3–48·7) | 1·01 (0·87–1·18) | 1·01 (0·87–1·17) |
| South Canterbury (R) | 48·2 (29·4–67·0) | 1·17 (0·76–1·80) | 1·16 (0·78–1·73) | 46·7 (32·1–61·3) | 1·14 (0·84–1·56) | 1·15 (0·84–1·56) |
| Southern (U) | 50·4 (41·4–59·4) | 1·28 (1·00–1·62) | 1·31 (1·04–1·66) | 54·0 (48·0–60·0) | 1·28 (1·10–1·50) | 1·27 (1·09–1·48) |
| By rurality |  |  |  |  |  |  |
| Urban regions combined | 44·6 (41·9–47·3) | [Reference] | [Reference] | 45·1 (43·2–47·0) | [Reference] | [Reference] |
| Rural regions combined | 47·8 (43·6–52·0) | 1·06 (0·96–1·17) | 1·07 (0·96–1·18) | 47·4 (44·3–50·5) | 1·06 (0·99–1·15) | 1·04 (0·96–1·12) |

CFR = case-fatality rate; CI = confidence interval; DHB = district health board; R = rural region; RR = rate ratio; U = urban region

*Partly adjusted = adjusted for age

**Fully adjusted = adjusted for age and ethnicity

**Supplementary Table 4.** Age- and sex-adjusted rate ratios with 95% confidence intervals for subarachnoid haemorrhage and its 30-day case fatality by ethnicity and geographical regions of NZ.

|  | SAH incidence | | | | SAH case fatality | | | |
| --- | --- | --- | --- | --- | --- | --- | --- | --- |
|  | European/other | Māori | Pacific people | Asian | European/other | Māori | Pacific people | Asian |
| By DHBs |  |  |  |  |  |  |  |  |
| Northland (R) | 1·06 (0·88–1·27) | 1·63 (1·18–2·26) | 1·82 (0·71–4·63) | NA | 1·13 (0·93–1·36) | 0·95 (0·64–1·43) | 1·56 (0·68–3·60) | NA |
| Waitemata (U) | [Reference] | [Reference] | [Reference] | [Reference] | [Reference] | [Reference] | [Reference] | [Reference] |
| Auckland (U) | 0·89 (0·76–1·04) | 1·39 (0·97–2·00) | 1·32 (0·89–1·98) | 1·06 (0·76–1·48) | 1·06 (0·90–1·24) | 1·18 (0·77–1·80) | 0·91 (0·56–1·50) | 1·02 (0·69–1·50) |
| Counties Manukau (U) | 1·13 (0·97–1·31) | 1·72 (1·26–2·34) | 0·84 (0·56–1·24) | 1·02 (0·72–1·44) | 1·11 (0·95–1·30) | 1·00 (0·68–1·47) | 0·99 (0·62–1·59) | 1·25 (0·84–1·85) |
| Waikato (U) | 0·96 (0·83–1·11) | 1·45 (1·06–1·99) | 0·22 (0·05–0·92) | 0·84 (0·45–1·56) | 1·17 (1·01–1·36) | 1·22 (0·84–1·78) | 1·53 (0·36–6·45) | 0·79 (0·29–2·15) |
| Lakes (R) | 0·92 (0·72–1·17) | 1·53 (1·07–2·18) | 1·55 (0·55–4·37) | 1·03 (0·32–3·28) | 1·10 (0·85–1·41) | 1·06 (0·69–1·62) | 0·61 (0·11–3·35) | 1·33 (0·43–4·11) |
| Bay of Plenty (U) | 0·97 (0·82–1·15) | 1·34 (0·96–1·88) | 1·40 (0·50–3·93) | 1·88 (0·93–3·79) | 1·07 (0·90–1·26) | 1·15 (0·78–1·71) | 1·72 (0·77–3·83) | 1·08 (0·47–2·51) |
| Tairawhiti (R) | 1·00 (0·70–1·44) | 1·67 (1·14–2·46) | 1·04 (0·14–7·61) | 1·72 (0·24–12·43) | 1·41 (1·05–1·89) | 1·21 (0·78–1·85) | NA | 4·32 (2·93–6·38) |
| Hawke's Bay (R) | 0·98 (0·81–1·18) | 1·36 (0·95–1·95) | 0·85 (0·30–2·38) | 0·91 (0·28–2·90) | 1·15 (0·96–1·38) | 0·87 (0·54–1·40) | 0·64 (0·11–3·59) | 1·02 (0·30–3·53) |
| Taranaki (R) | 0·96 (0·78–1·19) | 0·89 (0·53–1·49) | 1·04 (0·14–7·56) | 0·54 (0·08–3·92) | 1·05 (0·85–1·31) | 0·74 (0·36–1·53) | NA | 3·72 (2·66–5·20) |
| MidCentral (R) | 0·90 (0·75–1·09) | 1·04 (0·68–1·59) | 1·63 (0·69–3·88) | 0·68 (0·25–1·86) | 1·19 (0·99–1·43) | 1·25 (0·78–2·00) | 1·01 (0·44–2·30) | 0·77 (0·13–4·35) |
| Whanganui (R) | 1·16 (0·91–1·48) | 1·20 (0·74–1·95) | 1·69 (0·41–7·01) | 1·01 (0·14–7·32) | 1·32 (1·08–1·62) | 1·54 (0·97–2·44) | 1·09 (0·36–3·29) | 1·57 (1·10–2·23) |
| Capital & Coast (U) | 0·75 (0·63–0·90) | 1·16 (0·78–1·75) | 1·32 (0·82–2·15) | 1·12 (0·70–1·80) | 1·02 (0·84–1·22) | 1·25 (0·79–1·98) | 0·55 (0·26–1·18) | 1·12 (0·64–1·98) |
| Hutt Valley (U) | 0·89 (0·72–1·10) | 1·44 (0·95–2·17) | 0·55 (0·23–1·30) | 0·54 (0·22–1·34) | 1·12 (0·89–1·40) | 0·88 (0·52–1·51) | 0·33 (0·06–1·94) | 1·17 (0·33–4·09) |
| Wairarapa (R) | 0·76 (0·54–1·06) | 0·78 (0·34–1·81) | 1·30 (0·18–9·51) | 1·78 (0·25–12·81) | 1·10 (0·75–1·60) | 0·78 (0·25–2·44) | 1·84 (1·13–2·98) | NA |
| Nelson Marlborough (R) | 1·07 (0·90–1·27) | 0·84 (0·46–1·53) | 1·31 (0·31–5·42) | 1·00 (0·24–4·08) | 1·29 (1·10–1·52) | 1·12 (0·59–2·14) | NA | 1·50 (0·47–4·81) |
| West Coast (R) | 1·12 (0·81–1·54) | 0·49 (0·12–1·99) | NA | NA | 1·23 (0·89–1·70) | 2·02 (1·30–3·14) | NA | NA |
| Canterbury (U) | 1·02 (0·90–1·16) | 0·96 (0·64–1·44) | 0·62 (0·26–1·46) | 1·00 (0·61–1·63) | 1·04 (0·91–1·19) | 1·26 (0·80–1·97) | NA | 1·60 (0·99–2·61) |
| South Canterbury (R) | 0·90 (0·69–1·17) | 1·36 (0·59–3·16) | NA | 1·28 (0·18–9·27) | 1·14 (0·87–1·47) | 1·63 (0·83–3·20) | NA | NA |
| Southern (U) | 1·08 (0·94–1·25) | 0·83 (0·51–1·33) | 0·49 (0·12–2·04) | 0·80 (0·32–1·98) | 1·30 (1·13–1·49) | 1·23 (0·75–2·04) | 1·06 (0·40–2·80) | 2·06 (1·10–3·89) |
| By rurality |  |  |  |  |  |  |  |  |
| Urban regions combined | [Reference] | [Reference] | [Reference] | [Reference] | [Reference] | [Reference] | [Reference] | [Reference] |
| Rural regions combined | 1·01 (0·94–1·08) | 1·02 (0·89–1·15) | 1·36 (0·90–2·03) | 0·77 (0·47–1·25) | 1·08 (1·01–1·16) | 0·94 (0·82–1·09) | 0·98 (0·60–1·61) | 1·18 (0·75–1·86) |

CFR = case-fatality rate; CI = confidence interval; DHB = district health board; NA = not applicable due to low number of (fatal) events; R = rural region; RR = rate ratio; SAH=subarachnoid haemorrhage; U = urban region

**Supplementary Table 5.** Comparison between the current study from New Zealand and the previous studies from Finland.

|  | Current study from New Zealand | Previous studies from Finland |
| --- | --- | --- |
| Datasets | Externally validated nationwide hospital discharge and cause-of-death data collections | Externally validated nationwide hospital discharge and cause-of-death data collections |
| Study regions | 20 district health boards covering the whole country | 5 university hospital catchment areas covering the whole country |
| Study years | 2001–2018 | 1998–2017 |
| Follow-up in cumulative person-years | 78,187,500 | 106,510,337 |
| Number of SAH cases | 5,371 | 9.443 |
| Validity of SAH cases | Sensitivity 95%  PPV 85% in all patients, 92% among hospitalized patients and 96% among patients admitted to the neurosurgery | PPV 99·8% among cases admitted to the neurosurgical unit of Helsinki University Hospital |
| Nationwide crude SAH incidence per 100,000 person-years | 6·9 | 8·9 |
| Nationwide age-standardized SAH incidence per 100,000 person-years* | 8·2 | 9·1 |
| Nationwide average annual change in SAH incidence. % (95% CIs)** | 2·2% (1·7–2·7%) per year | 2·6% (2·3–2·9%) per year |
| Maximum regional SAH incidence difference** | 96% | 40% |
| Nationwide 30-day case-fatality rate | 46% | 39% |
| Nationwide average annual change in SAH case fatality*** | 1·3% (0·2%–2·3%) decline per year | 1·8% (1·1–2·6%) decline per year |
| Maximum regional SAH case-fatality difference**** | 126% | 52% |

*standardized to the age structure of the European Standard Population in 2013.

**Based on the age-, sex- and region-adjusted incidence rate ratios

***Based on the age- and sex-adjusted odds ratios

****Based on the age-, sex- and year-adjusted odds ratios

**Supplementary Figure 1A–G.** Annual autopsy rates for all out-of-hospital deaths (facility code of death was 'unknown' or '9990') in NZ by age groups, sex and ethnic groups between 2001 and 2018. Data were requested from the NZ Ministry of Health and are based on the Mortality Collection.

1. Overall

1. Men

1. Women

1. European/others

1. Māori

1. Pacific peoples

1. Asian
